# Procalcitonin to guide antibiotic use during the first wave of COVID-19 in English and Welsh hospitals: integration and triangulation of findings from quantitative and qualitative sources

**DOI:** 10.1136/bmjopen-2024-093210

**Published:** 2025-08-08

**Authors:** Josie Henley, Lucy Brookes-Howell, Philip Howard, Neil Powell, Mahableshwar Albur, Stuart E Bond, Joanne Euden, Paul Dark, Detelina Grozeva, Thomas P Hellyer, Susan Hopkins, Martin Llewelyn, Wakunyambo Maboshe, Iain J McCullagh, Margaret Ogden, Philip Pallmann, Helena K Parsons, David G Partridge, Dominick Shaw, Bethany Shinkins, Tamas Szakmany, Stacy Todd, Robert M West, Emma Thomas-Jones, Enitan Carrol, Jonathan Sandoe, On behalf of the PEACH Study Group

**Affiliations:** 1 School of Social Sciences, Cardiff University, Cardiff, UK; 2 College of Biomedical and Life Sciences, Cardiff University Centre for Trials Research, Cardiff, Wales, UK; 3 School of Healthcare, University of Leeds, Leeds, UK; 4 Medicines and Pharmacy, NHS England North East and Yorkshire, Leeds, West Yorkshire, UK; 5 Royal Cornwall Hospitals NHS Trust, Truro, UK; 6 Microbiology, NHS North Bristol NHS Trust, Bristol, England, UK; 7 Mid Yorkshire Hospitals NHS Trust, Wakefield, UK; 8 Division of Immunology, Immunity to Infection and Respiratory Medicine, The University of Manchester, Manchester, England, UK; 9 Translational and Clinical Research Institute, Newcastle University, Newcastle upon Tyne, UK; 10 Critical Care Department, Royal Victoria Infirmary, Newcastle upon Tyne, England, UK; 11 UK Health Security Agency, London, England, UK; 12 Global Health and Infectious Diseases, University of Sussex, Brighton, UK; 13 Translational and Clinical Research Institute, Newcastle University, Newcastle upon Tyne, England, UK; 14 Public and Patient Involvement Representative, Cardiff, UK; 15 Sheffield Teaching Hospitals NHS Foundation Trust, Sheffield, UK; 16 NIHR Leicester Biomedical Research Centre Respiratory Diseases, Leicester, East Midlands, UK; 17 University of Leeds Leeds Institute of Health Sciences, Leeds, West Yorkshire, UK; 18 University of Warwick Division of Health Sciences, Coventry, UK; 19 Department of Anaesthesia, Intensive Care and Pain Medicine, Division of Population Medicine, Cardiff University, Cardiff, UK; 20 Critical Care Directorate, Aneurin Bevan University Health Board, Newport, UK; 21 Liverpool University Hospitals NHS Foundation Trust, Liverpool, England, UK; 22 Department of Clinical Infection, Microbiology and Immunology, Institute of Infection, Veterinary and Ecological Sciences, University of Liverpool, Liverpool, UK; 23 Department of Microbiology, Leeds General Infirmary, Leeds, England, UK; 24 Healthcare Associated Infection Group, University of Leeds Leeds Institute of Medical Research, Leeds, England, UK; 25 All members of the PEACH study group are included in online supplemental file 1

**Keywords:** COVID-19, Antibiotics, Protocols & guidelines, Diagnostic microbiology, Methods

## Abstract

**Abstract:**

**Aim:**

To integrate the quantitative and qualitative data collected as part of the PEACH (Procalcitonin: Evaluation of Antibiotic use in COVID-19 Hospitalised patients) study, which evaluated whether procalcitonin (PCT) testing should be used to guide antibiotic prescribing and safely reduce antibiotic use among patients admitted to acute UK National Health Service (NHS) hospitals.

**Design:**

Triangulation to integrate quantitative and qualitative data.

**Setting and participants:**

Four data sources in 148 NHS hospitals in England and Wales including data from 6089 patients.

**Method:**

A triangulation protocol was used to integrate three quantitative data sources (survey, organisation-level data and patient-level data: data sources 1, 2 and 3) and one qualitative data source (clinician interviews: data source 4) collected as part of the PEACH study. Analysis of data sources initially took place independently, and then, key findings for each data source were added to a matrix. A series of interactive discussion meetings took place with quantitative, qualitative and clinical researchers, together with patient and public involvement (PPI) representatives, to group the key findings and produce seven statements relating to the study objectives. Each statement and the key findings related to that statement were considered alongside an assessment of whether there was agreement, partial agreement, dissonance or silence across all four data sources (convergence coding). The matrix was then interpreted to produce a narrative for each statement.

**Objective:**

To explore whether PCT testing safely reduced antibiotic use during the first wave of the COVID-19 pandemic.

**Results:**

Seven statements were produced relating to the PEACH study objective. There was agreement across all four data sources for our first key statement, ‘During the first wave of the pandemic (01/02/2020-30/06/2020), PCT testing reduced antibiotic prescribing’. The second statement was related to this key statement, ‘During the first wave of the pandemic (01/02/2020-30/06/2020), PCT testing *safely* reduced antibiotic prescribing’. Partial agreement was found between data sources 3 (quantitative patient-level data) and 4 (qualitative clinician interviews). There were no data regarding safety from data sources 1 or 2 (quantitative survey and organisational-level data) to contribute to this statement. For statements three and four, ‘PCT was not used as a central factor influencing antibiotic prescribing’, and ‘PCT testing reduced antibiotic prescribing in the emergency department (ED)/acute medical unit (AMU),’ there was agreement between data source 2 (organisational-level data) and data source 4 (interviews with clinicians). The remaining two data sources (survey and patient-level data) contributed no data on this statement. For statement five, ‘PCT testing reduced antibiotic prescribing in the intensive care unit (ICU)’, there was disagreement between data sources 2 and 3 (organisational-level data and patient-level data) and data source 4 (clinician interviews). Data source 1 (survey) did not provide data on this statement. We therefore assigned dissonance to this statement. For statement six, ‘There were many barriers to implementing PCT testing during the first wave of COVID-19’, there was partial agreement between data source 1 (survey) and data source 4 (clinician interviews) and no data provided by the two remaining data sources (organisational-level data and patient-level data). For statement seven, ‘Local PCT guidelines/protocols were perceived to be valuable’, only data source 4 (clinician interviews) provided data. The clinicians expressed that guidelines were valuable, but as there was no data from the other three data sources, we assigned silence to this statement.

**Conclusion:**

There was agreement between all four data sources on our key finding ‘during the first wave of the pandemic (01/02/2020-30/06/2020), PCT testing reduced antibiotic prescribing’. Data, methodological and investigator triangulation, and a transparent triangulation protocol give validity to this finding.

**Trial registration number:**

ISRCTN66682918.

**STRENGTHS AND LIMITATIONS OF THIS STUDY**

- Methodological triangulation, incorporating diverse data collection techniques, provides a comprehensive understanding of procalcitonin testing and antibiotic use during the first wave of COVID-19.
- Data triangulation, using qualitative and quantitative data across different levels (patients, clinicians, organisations), ensures a multifaceted analysis of the research problem.
- Investigator triangulation, involving analysts from varied backgrounds (qualitative, quantitative, clinical and patient and public involvement), enriches the analysis and improves validity.
- There is no accepted standardised method or high-level guidance for triangulation, which may introduce variability in the process.
- Despite efforts to ensure transparency and rigour, there remains a risk of bias in the triangulation process.

## Introduction

 Antimicrobial stewardship (AMS) is a term that is widely used to describe the systems and processes in place in an organisation to help optimise the use of antibiotics. AMS is vital because overuse and misuse of antibiotics are common and are driving antimicrobial resistance. The emergence and rapid global spread of SARS-CoV-2 impacted on antibiotic prescribing and disrupted usual AMS practices.^1^,^3^

Early in the COVID-19 pandemic, there was concern that antibiotic prescribing would surge, aggravating existing antimicrobial resistance problems and increasing adverse reactions. For hospitalised patients in England, this was a genuine concern as subsequent analysis showed that the rate of antibiotic prescribing increased in April 2020 and WHO classified ‘Watch’ antibiotic use increased, although lock-downs and reduced hospital activity may have contributed to an overall reduction in antibiotic use in this period.^3^ Over 70% of COVID-19 patients were prescribed antibiotics despite low (7%) rates of secondary bacterial infection.^1 4 5^ A distinction is drawn between bacterial coinfection (present at presentation) and secondary bacterial infection (which develops after presentation) in patients with COVID-19.^4^

Increasing attention is being paid to the role of diagnostic stewardship in antimicrobial prescribing. Diagnostic stewardship has been described as the processes of modifying the ordering, performing and reporting of diagnostic tests to improve the diagnosis of infection but is a complex process that involves entire multidisciplinary teams.^6^ There has been recognition of both the harms and benefits that diagnostic testing can provide in the context of infection.^6 7^ The aim of diagnostic stewardship is “to improve patient care by promoting accurate and timely diagnosis and thereby increasing appropriate antimicrobial use while reducing antimicrobial resistance”^5^ and is therefore, a crucial component of AMS.

Procalcitonin (PCT) is an inflammatory biomarker that increases in bacterial infection and was being used as a test for bacterial infection prior to COVID-19.^8 9^ Many hospitals introduced measurement of PCT during the first wave of the pandemic, in an attempt to improve the diagnosis of secondary bacterial infection and hence use of antibiotics.^10^ The Procalcitonin: Evaluation of Antibiotic use in COVID-19 Hospitalised patients (PEACH) study has used a mixed methods approach to evaluate whether the use of PCT testing to guide antibiotic prescribing safely reduced antibiotic use among patients admitted to acute UK National Health Service (NHS) hospitals and who had a diagnosis of COVID-19 during the first wave of the pandemic.^10^,^14^

Triangulation is a term used to describe the processes by which the output from different research approaches can be integrated to produce a more complete picture of the research findings.^15 16^ A triangulation protocol enables assessment of the degree of agreement (convergence), complementarity or contradiction (dissonance) between different approaches.^15^ In this work, we aimed to assimilate and compare the outputs from the different PEACH work packages.

## Methods

### Study design

We used a triangulation protocol technique^15^,^17^ to integrate quantitative and qualitative data collected as part of a programme of studies to explore whether PCT testing safely reduced antibiotic use during the first wave of the COVID-19 pandemic.^13^

### Setting and participants

Data source 1: antimicrobial pharmacists and doctors from acute NHS hospitals in England and Wales (n=148).^10^

Data source 2: antibiotic dispensing in English and Welsh hospitals (n=121).^11^

Data source 3: hospital patients with a positive COVID-19 test in England and Wales (n=6089).^14^ Inclusion criteria: patients ≥16 years, admitted to participating Trusts/Health Boards and with a confirmed positive COVID-19 test between 1 February 2020 and 30 June 2020.

Data source 4: clinicians from NHS Trusts/Health Boards in England and Wales who worked during the first wave of the COVID-19 pandemic (defined as March to June 2020) with maximum variation across role and hospital site (n=29).^18^

### Data collection

Data source 1 was a web-based survey to gather information about use of PCT for AMS purposes during the first wave of COVID-19 in England and Wales.^10^ The survey was piloted, refined and then distributed through UK antimicrobial pharmacist networks and the UK Clinical Pharmacy Network.

Data source 2 involved data on antibiotic usage (provided by Rx-Info Ltd), hospital activity (provided by Public Health England and Public Health Wales) and PCT usage (gathered through the web-based survey as described above for data source 1).^11^

Data source 3 was quantitative patient-level clinical data identified from institutional databases and patient medical records from 11 sites, including antibiotics used during treatment episode, length of hospital and intensive care unit (ICU) stay, and mortality rates.^14^ We used propensity score matching in data source 3 to ensure an even distribution of important confounders between the tested groups. Propensity-score matching was used to reduce the potential differences between the ‘tested’ (ie, PCT test at baseline) and ‘untested’ patients (ie, no PCT test at baseline) as described in the main paper for this data source.^14^

Data source 4 was individual semistructured qualitative interviews carried out remotely.^18^ Participant information sheets and expressions of interest for the qualitative interviews were disseminated to managers of departments within six of the 11 sites included in data source 3 via e-mail by the site principal investigators. Sites were invited to participate based on their routine clinical use of PCT testing before and during the pandemic to ensure sites were included that did not use PCT, that did use PCT and that introduced PCT during the first wave.^14^ Prospective sampling was conducted with an aim to recruit participants with varied experience and roles within each site. Potential participants volunteered to participate and returned a consent to contact form and were contacted by the PEACH qualitative researcher to arrange an interview. In the UK, and in accordance with the General Data Protection Regulation 2016/679 (GDPR), researchers cannot record personal information including contact details from a third party without consent of the individual.

### Analysis

We used three triangulation approaches: 1. Methodological triangulation with multiple data collection techniques (interviews, survey, institutional databases and records, and patient medical records); 2. Data triangulation (using text and numbers, and focused on groups at different levels: patient, clinician and organisation); and 3. Investigator triangulation (using multiple analysts with different backgrounds including qualitative, quantitative, clinical and patient and public involvement (PPI)).

Data from the four data sources were initially analysed individually in four separate analyses: data source 1 (led by NP and PH), data source 2 (led by ML and DG, analysed by DG/PP/RW), data source 3 (led by JS, analysed by DG/PP/RW) and data source 4 (led and analysed by JH and LBH). The methods used to collect and analyse data are reported elsewhere.^10^,^1418^ We then used a triangulation protocol to compare and integrate all four data sources.^17^ We have attempted to clearly articulate the triangulation process in order to allow transparency and contribute to the validity of our findings.

Step 1: we created a triangulation matrix in Excel with a separate column for each of the four data sources. The triangulation matrix was stored in a shared Teams space to allow representatives from the four data sources to each note down the main findings for that data source. This process happened over a period of months as the analysis of some of the datasets was complete before others. For example, the analyses for data sources 1 and 2 were completed first and results published while the analyses for 3 and 4 were ongoing.

Step 2: an online interactive discussion meeting took place (12.07.23) with different analysts (qualitative, quantitative, clinical) to discuss the key findings in the matrix and attempt to look for areas where findings from the different data sources could be grouped or related to each other. We then presented the key findings as a series of statements which related to the main study objective, to assist with comparison across data sources. The statements were discussed and refined by the team until consensus was reached. The matrix continued to evolve through a process of discussion and reflection. To allow for timely completion of the triangulation exercise, we began to fill in the matrix and discuss the statements while the quantitative analysis (data source 3) was being refined and finalised with these results being confirmed in the matrix last.

We discussed the possibility of including health economics data^12 19^ in the triangulation process (12.07.23) but the team felt that this data source provided specific data on health economic outcomes, would be silent for other key findings and would not therefore add value to our key questions.

Step 3: the next step was convergence coding. We took each statement in turn to assess where findings from each data source had agreement (convergence), partial agreement (complementarity), dissonance (conflicting findings) or silence (only one data source contributing).^17^ An assessment of this was noted in the final column of the matrix. This was discussed and consensus agreed in a further two remote interactive meetings, between qualitative, PPI and clinical researchers (10.08.23), and qualitative, quantitative and clinical researchers (15.09.23), to obtain a consensus about the relationship between findings. The triangulation methodology used in this study differed from those of Farmer *et al*^16^ and Tonkin-Crine *et al*,^17^ as data sources were compared pairwise in those studies. In our study, we compared all four data sources for each statement, where data were available. Some of the data sources were not able to answer specific questions as the research question had not been asked during the study.

Step 4: the last step was interpretation of the matrix, complete with convergence coding, to provide a narrative for each statement. We present this interpretive analysis below, and, where appropriate, have added evidence in the form of data quotes to illustrate the points.

### Patient and Public Involvement:

Patient and public representatives were involved during all stages of this research, as part of the overall PEACH study.

## Results

### Descriptive data

Four sets of data were gathered as part of the PEACH project, consisting of three quantitative data sources and one qualitative. Data source 1 comprised quantitative survey results from antimicrobial pharmacists and doctors from 148 of 151 (98.0%) acute NHS hospitals in England and Wales.^10^ Data source 2 was quantitative data of PCT use, antibiotic use and hospital activity of 105 English NHS Trusts and 16 Welsh NHS Hospitals.^11^ Data source 3 was quantitative data from 6089 individuals across 11 hospital sites in England and Wales.^12^ Data source 4 was qualitative interviews with 29 clinicians across six NHS Trusts/Health Boards in England and Wales, which were a subset of the sites in data source 3.^18^

### Interpretive analysis

We compared results from the four data sources for seven key statements related to PCT testing, antibiotic prescribing and guidelines. The coding matrix is summarised in table 1 below, and more detail is given following this summary table (full matrix included in online supplemental information). The summary table format is based on Parisi *et al*.^20^

**Table 1:** Summary table of triangulation coding matrix, based on Parisi *et al*^20^

Statement	Data source 1: survey (Quan)	Data source 2: organisational-level data (Quan)	Data source 3: patient-level data (Quan)	Data source 4: interviews (Qual)	Convergence coding
1	During the first wave of the pandemic (01/02/2020-30/06/2020), PCT testing reduced antibiotic prescribing	Agree	Agree	Agree	Agree	Agreement
2	During the first wave of the pandemic (01/02/2020-30/06/2020), PCT testing safely reduced antibiotic prescribing	No data	No data	Agree	Partial agreement	Partial agreement
3	PCT was not used as a central factor influencing antibiotic prescribing	No data	Agree	No data	Agree	Agreement
4	PCT testing reduced antibiotic prescribing in ED/AMU	No data	Agree	No data	Agree	Agreement
5	PCT testing reduced antibiotic prescribing in ICU	No data	Disagree	Disagree	Agree	Dissonance
6	There were many barriers to implementing PCT testing during the first wave of COVID-19	Partial agreement	No data	No data	Agree	Partial agreement
7	Local PCT guidelines/protocols were perceived to be valuable	No data	No data	No data	Agree	Silence

AMU, acute medical unit; ED, emergency department; ICU, intensive care unit; PCT, procalcitonin.

### During the first wave of the pandemic (01/02/2020-30/06/2020), PCT testing reduced antibiotic prescribing

This first statement is our key finding, and agreement was found between all four datasets.

#### Data source 1: Survey: Agree

There was a perceived value of PCT by the majority of respondents of the survey. 78 of 114 respondents (68.4%) responded “yes somewhat” or “yes very much” to the question of whether PCT had a positive effect on controlling antibiotic overuse in COVID-19. 12/114 (10.5%) responded with “probably not” or “not at all”, and 24/114 (21.1%) were unsure.^10^

#### Data source 2: Organisational level data: Agree

The introduction of PCT in emergency departments/acute medical admission units (ED/AMU) was associated with an initial statistically significant decrease in total antibiotic use of 1.08 (95% CI: 0.36 to 1.81) defined daily doses (DDDs) of antibiotic per admission per week per NHS Trust.^11^

#### Data source 3: Patient level data: Agree

PCT use was associated with reduced days of early antibiotics (within the first 7 days of a positive COVID-19 test) and reduced total days of antibiotic treatment. PCT testing at baseline was associated with a statistically significant average reduction in the duration of early antibiotics of 0.43 (95% CI: 0.22 to 0.64) days. PCT testing was also associated with an effect on total antibiotic prescribing during the hospital stay, with an average reduction of 0.72 (95% CI: 0.06 to 1.38) days.^14^

#### Data source 4: Qualitative interviews: Agree

Clinicians in hospitals where PCT was used previously or introduced during the first wave of the pandemic reported that the PCT test contributed to decision-making about antibiotic prescribing.^18^ They predicted that unnecessary antibiotic doses would have been reduced where the test was carried out. The stopping of antibiotics early was attributed to PCT results. Participant identifiers are the same as were used in the full paper on this source.^18^

Yes, yes, I think I can fairly, comfortably, and confidently make that statement, that PCT does reduce the use of antibiotics. It may not reduce the starting of antibiotics because you don’t have the results when you have to start but it makes it easier to stop them after two days rather than letting it run the course of five days.

P04 Non-consultant physician

Used PCT before and during first wave

I think, from practice, I think the general consensus was that actually this [PCT testing] is the easiest way for us to differentiate and actually treat our patients properly and correctly. Without it, are we going to be treating every single patient with steroids and antibiotics? In which case that’s probably what would have happened and would be continuing to happen if we didn’t have the procalcitonin.

P05 Pharmacist

Used PCT before and during first wave

In hospitals where PCT was not used, participants reported that antibiotics were being prescribed more readily during the first wave of the pandemic, due to the lack of available treatments for COVID-19, often despite clinicians’ better judgement.

Everyone was getting antibiotics like smarties just in case.

P07 Consultant

Did not use PCT before or during first wave

We are back in last year… I would still give her antibiotics… Now maybe I would culture and watch. But then, I would have still given antibiotics.

P08 Consultant

Did not use PCT before or during first wave

### During the first wave of the pandemic (01/02/2020-30/06/2020), PCT testing safely reduced antibiotic prescribing

This statement is related to our key finding, with the addition of the word ‘safely’, as it was part of our original aims to assess whether any increased mortality was associated with the reduction in antibiotic prescribing. Partial agreement was found between two of the datasets: data sources 3 (patient-level data) and 4 (qualitative clinician interviews). We have no data from data sources 1 or 2 for this statement. Data source 3 clearly agrees with the statement; whereas, data source 4 was in partial agreement as some clinicians were not confident that reducing antibiotic prescribing using PCT testing was harm-free. We have therefore assigned partial agreement to this statement. This partial agreement is based on clinicians’ opinions. It should be noted that the clinicians who expressed concern over PCT use suggested that more evidence was needed for the safety of the test. This was prior to the publication of findings from data source 3, the patient-level data, which does indicate that PCT testing was harm-free.

#### Data source 3: Patient level data: Agree

PCT testing was not associated with increased 30-day or 60-day mortality and was not associated with an increase in hospital or ICU length of stay.^14^ This indicates that there were no detectable detrimental effects of PCT testing in terms of these measured outcomes.

#### Data source 4: Qualitative interviews: Partial agreement

Most clinicians were positive about the use of PCT in guiding them to make antibiotic prescribing decisions. There was a divide between the majority who made the judgement that PCT had contributed safely to the reduction of antibiotic use within their hospital, and a minority who were more circumspect and would prefer to see evidence for the efficacy of PCT before it was used widely.^18^

Those who judged that the use of PCT contributed safely to the reduction in antibiotic prescribing included people who had previous experience with PCT, such as the pharmacist in the quote below, who attributed the successful use of PCT with COVID-19 to this familiarity.

I think it’s definitely been useful, and it has safely contributed to the management of antibiotics within our Health Board and in COVID patients, and I think part of that goes for the fact that we were already using it. We were already quite familiar with the process and how to interpret PCT levels, but obviously as the complexity of COVID-19, yes I think it has positively impacted the review of using antibiotics.

P15 Pharmacist

Used PCT before and during first wave

Those who saw PCT as safely guiding antibiotic prescribing decisions also included people new to PCT use, such as the consultant below.

I really think it [PCT] was a useful test, I really think it guided us through it [COVID-19 pandemic] in a safe way, and pragmatic way so we didn’t over-treat, and again, didn’t under*-*treat either, which is important too.

P24 Consultant

Introduced PCT during first wave

Conversely, there were clinicians who were wary of the risk of using a test designed for one purpose in another situation. Some clinicians believed that there was not enough evidence at the time and information to begin a roll-out of PCT use. This included the ED consultant below.

Because it seems to make sense and we use it for this and it shows bacterial infection in respiratory diseases, therefore maybe we should use it in cellulitis. And say whether that, you know, is it an insect bite? Is it a reaction, or is it a bacterial infection? Well, let’s do a procalcitonin. Whoa, hang on a minute, I’ve not seen any RCTs [randomised controlled trials] yet to look at the, whether procalcitonin accurately predicts bacterial infection in cellulitis. You just can’t assume it does. And I think that’s one of the things within hospital medicine… is that something finds a use case and then people try and extrapolate that to how it can be used elsewhere, because it’s proved useful, without necessarily having the evidence to back it up.

P02 Consultant

Used PCT before and during first wave

Some clinicians indicated that they would not be happy with PCT being used outside of a trial setting unless this was evidence-based practice. They worried that there could be an over-reliance and that once a test is available, it is difficult to remove it. Therefore, the use of the test should be restricted and managed until the clinical trial evidence was published.

I think the only thing is kind of my disappointment that we seem to have implemented things like procalcitonin or certainly improved access to it, without necessarily an evidence base to support its use. And it’s very difficult to get people to de-adopt things that they believe in their gut… help them with clinical decision-making. De-adoption is more difficult than adoption, right, because stopping antibiotics is more difficult than starting them. And it would be great if we were to face, you know, another pandemic, that we, in our enthusiasm to do the right thing, didn’t step too far.

P26 Consultant

Did not use PCT before or during first wave

I am not sure if that is the right direction to have gone in because I am not sure the evidence is there that we should be doing [PCT testing]. So, I think people have got comfortable with using it [PCT], but I worry then that they are going to start using it where it’s not applicable… starting to use it where we haven’t before and the evidence doesn’t support it and that’s then not the right route to go down, is it? And so, I think trying to rein that back in and keep it just as a ‘this is for, at the moment, these are the scenarios that we are comfortable and happy with using and the evidence is there for’ and not allowing it just to be used. It is very difficult the way our lab is set up, it doesn’t really allow us to restrict things very well it doesn’t seem.

P22 Consultant

Introduced PCT during first wave

### PCT was not used as a central factor influencing antibiotic prescribing

For the statement about PCT not being a central factor in influencing the decision about whether to prescribe antibiotics, we had no data from either data source 1 (survey) as the question was not asked, or data source 3 (patient-level data). There was agreement between data sources 2 and 4. We have therefore assigned agreement to this statement.

#### Data source 2: Organisational level data: Agree

Although there was an initial significant drop in organisational prescribing, this declined over time. This effect was subsequently lost at a rate of 0.05 (95% CI: 0.02 to 0.08) DDDs per admission per week per Trust. Similar effects were found for first-line antibiotics prescribed for community-acquired pneumonia and for analysis restricted to COVID-19 admissions.^11^

#### Data source 4: Qualitative interviews: Agree

During the first wave, there was a lot of confusion and rapidly changing advice around tests and treatments. Some clinicians reported that the tests contributed very little to the decision-making around antibiotic prescriptions, as these decisions were based on clinical judgement.^18^

I mean, we were enthusiastic about it [PCT] at the start, we still are. I’m not surprised at all about finding that severe COVID puts up a PCT because as I say, the immune system isn’t that specific. So, I think it’s something that’s incredibly useful, but it is not purely diagnostic in its own right. That’s how I would view it.

P17 Consultant

Introduced PCT during first wave

It would be part of a milieu of clinical stuff, you know, I definitely wouldn’t be making a decision just on the PCT, but it would contribute to that decision-making process.

P06 Non-consultant physician

Did not use PCT before or during first wave

I would never solely use the PCT value to make any recommendations to a consultant or a doctor on whether it [antibiotics] should be stopped. It would probably be there as a tool to help my decision-making… It provided part of the information that we needed for our decision-making but not all of it.

P15 Pharmacist

Used PCT before and during first wave

As clinicians gained more confidence in recognising COVID-19, they became more confident to withhold antibiotics. This might have led to a familiarity with PCT as it became a routine part of the diagnostic process, or to less reliance on PCT testing.

I’d say my familiarity with it is better; therefore, my confidence with it is better. It’s like any new test that comes in, you just get a feel, you get that feel for it don’t you? You just get used to using it until it becomes like it is now where you would never not want to have it.

P13 Consultant

Used PCT before and during first wave

Some clinicians expressed difficulty in using PCT due to a lack of information or training on its use.

I might have discarded that [PCT result], and I would have probably discarded that quicker, than I might have done other tests. Because I think I would have looked at it and probably said, I don’t fully understand what that means, so I’m just going to disregard it, at the beginning.

P21 Non-consultant physician

Introduced PCT during first wave

### PCT testing reduced antibiotic prescribing in ED/AMU

For the statement about antibiotic prescribing reduction specifically in ED/AMU, we have no data from data sources 1 or 3. There was agreement between data sources 2 and 4. We have therefore assigned agreement to this statement.

#### Data source 2: Organisational level data: Agree

Introduction of PCT in ED/AMU was associated with an initial statistically significant decrease in total antibiotic use as reported above for Statement 1.^11^

#### Data source 4: Qualitative interviews: Agree

Clinicians in EDs found that PCT use was more widespread than in other parts of the hospital.^18^ This was partly due to a higher likelihood of antibiotics being prescribed in this setting. There was a heightened anxiety around the unknown infection at the beginning of the pandemic. This led to a need for more evidence for clinicians to be reassured in stopping antibiotics. EDs had a reputation for prescribing antibiotics to patients on admission, and clinicians saw their role as providing evidence for de-escalation. PCT was seen as a useful tool for providing this evidence.

The ED gets a bit of a bad rep for giving everyone broad-spectrum antibiotics as soon as they come in. And our role, to balance that up, is to make sure, our role in good antimicrobial stewardship is to think carefully about starting them, and to make sure we do the tests and investigations that allows to stop them and de-escalate… to make sure we send off procalcitonin, we send off blood cultures, we send off urine MC+S [microscopy, culture and sensitivity], we send off sputum, whatever we can get to sample and culture, that’s our job to make sure that’s gone, to enable those who are looking after the patient at 48 and 72 hours to do that review, see if they could be improving, see if they’re eating and drinking and can make a switch to orals. And see if we can de-escalate those antibiotics.

P02 Consultant

Used PCT before and during first wave

Really getting them trying to shut it [antibiotic prescribing] down and give people the confidence to say no, don’t worry, but very hard for the ED consultants to… they needed that reassurance I think to be able to not [prescribe antibiotics]. I think the PCT very much helped with that as well.

P22 Consultant

Introduced PCT during first wave

#### PCT testing reduced antibiotic prescribing in ICU: Disagree

For the finding about reducing antibiotic prescribing in the ICU, we had no data from data source 1. There was disagreement between data sources 2, 3 and 4: 2 and 3 both showed no significant prescribing reduction associated with ICU, while data source 4 (qualitative clinician interviews) suggested agreement with the statement. We therefore assigned dissonance to this statement.

### Data source 2: Organisational level data: Disagree

In ICU settings, PCT was not associated with any statistically significant change in antibiotic use.^11^

### Data source 3: Patient level data

There was no statistically significant association between antibiotic prescribing in patients admitted early to ICU and baseline PCT testing.^14^

### Data source 4: Qualitative interviews: Agree

Clinicians spoke about how PCT was used for reassurance purposes in ICU. As with ED, it was considered a useful tool to enable reduction of antibiotic use. Use of PCT in the ICU setting was seen as effective in ruling out bacterial infections and streamlining treatment, focusing solely on managing COVID-19-related issues. This was attributed to the level of sickness in the patient population in these settings.^18^

Mainly in ITU [intensive therapy unit], because ITU patients were really sick. So, when we were checking procalcitonin and we were like, “Okay, procalcitonin is negative.” So, it’s all just COVID going on. Because with COVID, I felt everybody was just feeling helpless, you’ve done this, you’ve done this, patients are not getting better. And then you’re like, “Oh, we’ve ruled out now bacterial infections as well, we have checked procalcitonin, we’ve given antibiotics.” So, it did kind of help because then you were just dealing with COVID, and just getting them on antibiotics, or changing one antibiotic after the other is not going to help. So, it did help [to reduce antibiotic prescriptions], mainly in the sicker patients.

P03 Non-consultant physician

Used PCT before and during first wave

For me in intensive care it helps me to stop the antibiotics very early on… We saved a lot of money with reduction of antibiotic usage which was obviously well received by everyone, reduction in antibiotics is obviously going to help the reduction resistance for organisms to develop antibody resistance… There was discussion about should we stop using the procalcitonin because it’s expensive etc. I don’t see that conversation ever coming again because we did, it’s pretty much engraved in our culture, really, and pretty much all the other nearby Health Boards have started using the procalcitonin on the back of COVID-19 as well.

P12 Consultant

Used PCT before and during first wave

In some hospitals, PCT was regularly used in ICU before the pandemic and clinicians were familiar with its use. This might explain the dissonance in these findings, as if PCT was in regular use in ICU, then antibiotic prescribing might already have been streamlined pre-COVID-19.

We were using it for many years before COVID-19 and I always found it positive and my colleagues always found it positive, especially during the winter time with the flu pneumonitis patients, and with COVID-19 it’s even more discussed in our meetings, really. So, it’s not unusual at all for our colleagues who’re just doing the handover of the patients to say the procalcitonin value etc. and the need to stop antibiotics or just change the antibiotics. So, I do think it’s changed for the better on the back of COVID-19, more and more people have started using it, it’s not just me but everyone around me has started using procalcitonin.

P12 Consultant

Used PCT before and during first wave

### There were many barriers to implementing PCT testing during the first wave of COVID-19

For the statement about barriers to implementing PCT testing, we have no data from data sources 2 or 3. There was partial agreement between data sources 1 and 4 in elements of this statement. Data source 1 indicates that guidelines were available, but not everywhere. However, it does not tell us how the guidelines were used or whether there were barriers to PCT use. Data source 4 goes into details about how the guidelines were used and what barriers people experienced. We therefore assigned partial agreement to this statement.

#### Data source 1: Survey: Partial agreement

55 of 114 (48.2%) of respondents reported that their organisation had a guideline for PCT use.^10^

#### Data source 4: Qualitative interviews: Agree

During the first wave of the pandemic, clinicians reported a lot of confusion and rapidly changing advice and guidelines around tests and treatments, meaning that guidelines were not always followed.^18^ There was therefore chaotic implementation of PCT testing, with some clinicians being aware of guidelines and others not, and with guidelines being followed differently in different parts of the hospital. For example, some hospitals had PCT as a standard test, but not others. Interviewees reported that PCT was “part of a COVID panel of bloods” for patients on initial investigations in some hospital wards, but not others. Some clinicians reported that PCT testing contributed very little to the decision-making around starting antibiotic prescriptions, because the results would not have been returned in time, or the patient would have moved into another part of the hospital.

The reported dearth of information about test interpretation implied a lack of sufficient guidance on PCT use. Difficulties with implementation of the test were attributed by many interviewees to under-confidence and training needs.

Initially, it was very hard because it was a new blood test, so we had no feel, and even some of the ITU consultants, they said, I’ve got no feel for what this means. Because a new… you normally have a reference range for a blood test, so you know, or you have a feel for it because there’s a normal, and then you know if something is a little bit up, and then you know what it is if it’s very, very up, and you should be very worried. But I think getting used to what was a high PCT and what was a just up PCT was quite hard as well.

P21 Non-consultant physician

Introduced PCT during first wave

Another barrier to implementing a new protocol was the lack of trust and lack of belief in PCT. This might have arisen from a lack of familiarity with the test, with its application to COVID-19, or with a level of scepticism about the veracity of the results, as with the clinician below.

I feel the main disadvantage is that in the back of everybody’s mind a lot of people have a little bit of doubt about procalcitonin. I think everybody has had a patient that has had a low procalcitonin but then later ended up having a bacterial infection. It only takes a small minority of patients to then, oh, you kind of have a little bit of doubt in the back of your mind.

P04 Non-consultant physician

Used PCT before and during first wave

### Local PCT guidelines/protocols were perceived to be valuable

For the statement about the perceived value of local guidelines and protocols, we have no data from data sources 1, 2 or 3. The only data are from data source 4. The clinicians expressed that guidelines were valuable, but as there were no data from the other three data sources, we have assigned silence to this statement.

#### Data source 4: Qualitative interviews: Agree

Clinicians who reported using the available guidelines said that they were helpful, especially due to COVID-19 being a new condition. This was particularly when the guidelines were very clear with respect to decision-making thresholds, and when they were readily available to access.^18^

It was very easy because every time you were starting to clerk a patient, the booklet had an extra paper for those patients. And it had many flowcharts and things, it had all the information anyone literally needed at that time, starting from when to start antibiotics, about oxygen, how to escalate, when to escalate, who to escalate… Every time I would go and look at a patient, I had this algorithm in my hand, so it was there… with every booklet you had that paper, so when you are clerking the patient you’re actually just holding that. You don’t have to spend extra time to look into guidelines, you just have it there at that time.

P03 Non-consultant physician

Used PCT before and during first wave

They were encouraging you to think whether the patient actually needed antibiotics, I think they gave some decent parameters in helping you decide whether they were necessary or not… any patient that was fairly seriously ill, it would recommend starting antibiotics. I remember the procalcitonin also being a factor in mainly helping you decide to stop anything, not helping you decide whether to start them, but if you started them, it helped you decide whether to stop them.

P04 Non-consultant physician

Used PCT before and during first wave

Those who did not have guidelines available said that they potentially would have helped. This included suggestions for the information the guidelines could contain and how they could be used.

I think if we had a combination of, you know, fine, initiate antibiotics at the front door, unless you know, but we review those antibiotics within 48 hours with a PCT test, typical features of COVID, please stop. I think the guidelines would have to be very explicit about that to help, or if it got to a point where you could turn around the test quickly enough for A&E or the admitting team not to initiate them but I don’t know how realistic that is in a sick group of people who are generally moving up quite promptly through A&E and the ground floor up to the wards. I think it could, but I just don’t know by how much.

P07 Consultant

Did not use PCT before or during first wave

The usefulness of guidelines was outlined by the following quote from an experienced consultant.

If you don’t have a guideline then you have ten different people prescribing ten different antibiotics and that’s not the right thing to do. So, having guidelines which are guided by the microbiologists who know the sensitivity of the resistance patterns of the organisms to antibiotics is very crucial… I think we should be lucky in that we have really good microbiologists who design guidelines for us so that we reduce unwanted variations in antibiotic use, and quite a lot of antibiotics they do change after a while because there’s a change in the sensitivity of the bugs. So, during the last nine years as a consultant, I’ve seen the antibiotic first choice for pneumonia, UTIs [urinary tract infections], they’ve been changed multiple times based on the resistance patterns to those antibiotics with organisms, really, so I think it useful to have guidelines, yes.

P12 Consultant

Used PCT before and during first wave

## Discussion

We found agreement across all our methods and data sources that PCT testing was associated with a reduction in antibiotic use during the first wave of the COVID-19 pandemic. There was agreement that PCT testing reduced antibiotic prescribing in EDs and AMUs but dissonance in terms of PCT use in the ICU. In the latter case, both sources of quantitative data found no association between PCT use in ICU and reduced antibiotic prescribing, while strong positive views were expressed in the qualitative work. Finding a reduction in antibiotic use is consistent with a systematic review examining the impact of PCT on antibiotic prescribing in respiratory tract infections before COVID-19.^21^ Several local reports have concluded that PCT use helped to reduce antibiotics during the first wave of the pandemic by comparing prescribing in those with a high PCT value to those with a low value.^22^,^25^ While these studies tell us about those who were tested, they do not confirm the value of testing per se. Several studies have compared patients with and without a PCT test and found a significant reduction in antibiotic use.^26^,^28^ Our findings of dissonance for an impact of PCT in the ICU setting were driven by a perceived value in qualitative work that was not reflected in our quantitative work. Our quantitative findings disagree with the published literature where two studies have reported antibiotic reductions in ICU during COVID-19.^22 26^

Not all our methods addressed the issue of the importance of PCT to influence antibiotic prescribing, but there was agreement that PCT was not a central factor and the severity of illness and vulnerability of the patient, for example, played a more important role. This is consistent with previous reports that found clinicians often continued antibiotics in the face of a low PCT.^22 24^

While guidelines were felt to be useful to guide use of PCT, there was inconsistent use of guidelines regarding PCT, antibiotics and COVID-19, and often a lack of guidelines altogether. This was attributed by the clinicians interviewed to the unprecedented rapidly changing situation of the pandemic, whereby any guidance was based on little evidence and outdated almost immediately. In this situation, clinicians reported relying on their own and colleagues’ judgement and expertise, while sharing the emerging evidence about the new infection. For more on this point, see our previous qualitative paper.^18^

In order to try to answer the question of whether PCT use during the first wave of the COVID-19 pandemic safely reduced antibiotic prescribing, we used a triangulation approach encompassing methodological and data triangulation. Methodological triangulation involved using more than one data collection technique: questionnaire data from hospitals, aggregated antibiotic usage data from hospital Trusts/Health Boards, patient-level clinical data and clinician interviews. This method has been previously used but not standardised. We followed a methodology presented by Farmer *et al*^16^ and Tonkin-Crine *et al*,^2^ in tabulating results for ease of comparison and convergence coding as a group, and transparency in the methodology. We followed the summary table format presented by Parisi *et al*^20^ to enable the differentiation between agreement (or not) with the statement by each data source and agreement (or not) between data sources. It should be noted that for some statements, not all data sources provided data.

### Reflection on triangulation process

We found that the triangulation process was useful in that the results from the different data sources added validity to our key finding of ‘During the first wave of the pandemic (01/02/2020-30/06/2020), PCT testing reduced antibiotic prescribing’. The agreement between all data sources for this finding provides strong evidence that our question has been answered in the positive. In comparing the results, we found information that might otherwise have been missed. We also noted an area where our results did not match other published results (PCT testing in ICU settings), which indicates further study is needed. It was useful and interesting to note the differences in perspectives from the different PEACH study work packages and data capture and analysis techniques. Using a template for the methodology helped in clarifying roles and tasks within the team.

### Strengths and Limitations

Strengths of triangulation include an enhancement of the robustness, validity and depth of research findings. Using methodological, data and investigator triangulation together amplifies the strengths of each approach. The comprehensive data collection, multilevel analysis and diverse expertise provide a robust and credible foundation for the research. Our triangulated approach enhances the credibility and utility of the research findings.

Although triangulation is seen by many as a crucial process to test agreement/disagreement between different approaches to asking the same research question, there is no accepted standardised method nor high-level guidance. There is a risk of bias in the process, which we sought to overcome by using a transparent and questioning methodology. Our research team has a broad range of expertise and includes non-clinicians and clinicians, including those who are sceptical about the value of PCT, but all have equipoise and would be willing to recruit into clinical trials.

When considering the source data, it should be noted that a study of viral pneumonia completed before COVID-19 found low mortality (1.6%) but that patients had a range of peak PCT values, indicating that viral infection can increase PCT.^29^ PCT did not distinguish well between pure viral pneumonia and bacterial coinfection in this study, but performed better as a prognostic marker.^29^ An association between raised PCT (>0.5 ng/mL) and poor prognosis in COVID-19 was noted early in the pandemic.^30^ This is relevant because PCT may have been carried out, either consciously or unconsciously, in more unwell patients, meaning that severity of illness is a potentially confounding factor in any analysis of PCT in COVID-19. This highlights why propensity score matching was necessary to reduce the risk of bias in the analysis of data source 3 and why there is a risk of bias in perspectives about the role of PCT.

In our study, we considered only hospitalised patients and did not consider which specific antibiotics were used before admission or take into account their consequences on long COVID-19. A limitation of the qualitative dataset is that participants volunteered to be interviewed, which exposes the data to selection bias.

### What this study adds

Using a multimethod/multidata source triangulation process, baseline PCT testing was associated with a reduction in antibiotic use in COVID-19 patients in hospital.

Agreement across all four data sources method gives validity to this finding.

Of the seven statements, we have three statements about antibiotic prescribing, two about areas of hospital where testing was performed (ED/ICU), and two related to guidelines.

## Conclusion

A triangulation process was successfully used to integrate the results from the quantitative and qualitative data from four individual work packages of the PEACH study.

There was agreement between all four data sources that PCT reduced antibiotic prescribing during the first wave of the pandemic. There was partial agreement with the statement concerning safety of PCT use, driven by some clinicians expressing concerns about the lack of data on safety prior to its introduction. There was agreement that PCT reduced antibiotic prescribing in EDs/AMUs but dissonance about its impact on ICUs. There was agreement that PCT testing was not a central factor in antibiotic prescribing decisions. Data, methodological and investigator triangulation, and a transparent triangulation protocol give validity to this finding.

## Supplementary material

10.1136/bmjopen-2024-093210 online supplemental file 1

10.1136/bmjopen-2024-093210 online supplemental file 2

10.1136/bmjopen-2024-093210 online supplemental file 3

## Data Availability

Data are available upon reasonable request.
