# Supplementary material for: Procalcitonin to guide antibiotic use during the first wave of COVID-19 in English and Welsh hospitals: integration and triangulation of findings from quantitative and qualitative sources
Source: BMJ Open. 2025 Aug 8;15(8):e093210. doi: 10.1136/bmjopen-2024-093210 (PMC12336465; doi:10.1136/bmjopen-2024-093210)
Supplement: online supplemental file 1 [file bmjopen-15-8-s001.docx]

**APPENDIX 1:PEACH Study Group**

**Co-Chief Investigators**

Jonathan Sandoe^1,2^

Enitan Carrol^3^

^1^ Department of Microbiology, The General Infirmary at Leeds, Leeds, UK

^2^ Healthcare Associated Infection Group, Leeds Institute of Medical Research, University of Leeds, Leeds, UK

^3^ Department of Clinical Infection, Microbiology and Immunology, Institute of Infection, Veterinary and Ecological Sciences, University of Liverpool, Liverpool, UK

**Coordinating Centre:**

*Study lead*: Emma Thomas-Jones,^1^

*Study Manager*: Joanne Euden,^1^

*Qualitative Researchers*: Lucy Brookes-Howell,^1^ Josie Henley,^2^

*Data Manager*: Wakunyambo Maboshe,^1^

*Co-lead Statistician*: Philip Pallmann,^1^

*Statistician*: Detelina Grozeva,^1^

*Database support*: Marcin Bargiel^1^

*Study Administrator*: Judith Evans^1^

^1^ Centre for Trials Research, College of Biomedical and Life Sciences, Cardiff University, Cardiff, UK

^2^ School of Social Sciences, Cardiff University, King Edward VII Avenue, Cardiff, CF10 3WA

**Research Team**

*Health Economics:* Edward Webb^1^ Rebecca Bestwick,^1^ Daniel Howdon,^1^ Natalie King,^1^ Bethany Shinkins (lead),^1,2^

*Co-lead Statistician*: Robert West,^1^

^1^ Leeds Institute for Health Sciences, University of Leeds, UK

^2^ Division of Health Sciences, University of Warwick, Coventry, UK

**Study Partners:**

*RX Info*: Colin Richman,^1^

*UK Health Security Agency (UKHSA)*: Sarah Gerver,^2^ Russell Hope,^2^ Susan Hopkins,^2^

*Public Health Wales*: Margaret Heginbothom,^3^

*NHS England*: Philip Howard,^4^

^1^ Rx-Info Ltd, Exeter Science Park, Exeter, EX5 2FN, UK

^2^ UK Health Security Agency (UKHSA), UK

^3^ Healthcare Associated Infection, Antimicrobial Resistance and Prescribing Programme, Public Health Wales, UK

^4^ NHS England and NHS Improvement, North-East and Yorkshire Region, UK

**Participating NHS Trusts:**

**Leeds Teaching Hospitals NHS Trust (lead Trust)**

*Principal Investigator*: Jonathan Sandoe,^1,2^

*Research Group (data collection – alphabetical order)* Claire Berry,^3^ Georgina Davis,^3^ Vikki Wilkinson,^3^

^1^ Department of Microbiology, The General Infirmary at Leeds, Leeds, UK

^2^ Healthcare Associated Infection Group, Leeds Institute of Medical Research, University of Leeds, Leeds, UK

^3^ Leeds Teaching Hospitals NHS Trust, Leeds, UK

**Liverpool University Hospitals NHS Foundation Trust**

*Principal Investigator*: Stacy Todd^1^

*Research Group (data collection – alphabetical order):* Eleanor Taylor-Barr,^1^ Mary Brodsky,^1^ Jo Brown^1^ Jenni Burns,^1^ Sharon Glynn,^1^ Alvyda Gureviciute,^1^ Megan Howard,^1^ Jennifer Kirkpatrick,^1^ Hannah Muphy,^1^ Emma Richardson,^1^ Deborah Scanlon,^1^ Claire Small,^1^ Graham Sweeney,^1^ Lisa Williams,^1^

^1^ Liverpool University Hospitals NHS Foundation Trust, Liverpool, UK

**Aneurin Bevan University Health Board**

*Principal Investigator*: Tamas Szakmany^1,2^

*Research Group (data collection – alphabetical order*): Evelyn Baker,^3^ Yusuf Cheema,^3^ Jill Dunhill,^3^ Charlotte Killick,^3^ Charlie King,^3^ Simran Kooner,^3^ Swyn Lewis,^3^ Maxine Nash,^3^ Owen Richardson,^3^ Jemma Tuffney,^3^ Clare Westacott,^3^ Sarah Williams,^3^

^1^ Critical Care Directorate, Aneurin Bevan University Health Board, Cwmbran, UK

^2^ Department of Anaesthesia, Intensive Care and Pain Medicine, Division of Population Medicine, Cardiff University, Cardiff, UK

^3^ Aneurin Bevan University Health Board, Cwmbran, UK

**Sheffield Teaching Hospital NHS Foundation Trust**

*Co-Principal Investigators:* David Partridge,^1^ Helena Parsons,^1^

*Research Group (data collection – alphabetical order*): Kay Cawthron,^1^ Yuen Kiu Tai,^1^ Thomas Newman,^1^ Megan Plowright,^1^ Helen Shulver,^1^ Anna Sivakova,^1^

^1^ Sheffield Teaching Hospitals NHS Foundation Trust, Sheffield, UK

**Royal Cornwall Hospitals NHS Trust**

*Principal Investigator*: Neil Powell^1^

*Research Group (data collection – alphabetical order*): Freddie Ayliffe,^1^ Emma Darke,^1^ Eve Fletcher,^1^ Fiona Hammonds,^1^ Gladys Marquez,^1^ Leanne Welch,^1^

^1^ Royal Cornwall Hospitals NHS Foundation Trust, Truro, UK

**Mid Yorkshire Teaching NHS Trust**

*Principal Investigator*: Stuart Bond^1^

*Research Group (data collection – alphabetical order*): Jade Lee-Milner,^2^

Joseph Spencer,^2^

^1^ Medicines Optimisation and Pharmacy Services, Pindersfield Hospital, Mid Yorkshire Teaching NHS Trust, Wakefield, UK

^2^ Mid Yorkshire Teaching NHS Trust, Wakefield, UK

**North Bristol NHS Trust, Bristol**

*Principal Investigator*: Mahableshwar Albur^1^

*Research Group (data collection – alphabetical order*): Rodrigo Brandao,^1^ Joshua Hrycaiczuk,^1^ Jack Stanley,^1^

^1^ North Bristol NHS Trust, Bristol, UK

**University Hospital Sussex NHS Foundation Trust**

*Principal Investigator*: Martin Llewelyn^1^

*Research Group (data collection – alphabetical order*): Elizabeth Cross,^2^ Daniel Hansen,^2^ Ethan Redmore,^2^ Abigail Whyte,^2^

^1^ Brighton and Sussex Medical School, University of Sussex and University Hospitals Sussex NHS Foundation Trust, Brighton UK

^2^ University Hospitals Sussex NHS Foundation Trust, Brighton, UK

**Newcastle-upon-Tyne Hospitals NHS Foundation Trust**

*Principal Investigators*: Tom Hellyer,^1,2^ Iain McCullagh,^1,2^

*Research Group (data collection – alphabetical order):* Benjamin Brown,^3^ Michele Calabrese,^3^ Cameron Cole,^3^ Jessica DeSousa,^3^ Leigh Dunn,^3^ Stephanie Grieveson,^3^ Arti Gulati,^3^ Elizabeth Issac,^3^ Ruaridh Mackay,^3^ Fatima Simoes,^3^

^1^ Critical Care Department, Royal Victoria Infirmary, The Newcastle-upon-Tyne Hospitals NHS Foundation Trust, Newcastle upon Tyne, UK

^2^ Translational and Clinical Research Institute, Newcastle University, Newcastle upon Tyne, UK

^3^ Newcastle-upon-Tyne Hospitals NHS Foundation Trust, Newcastle upon Tyne, UK

**Salford Royal NHS Foundation Trust**

*Principal Investigator*: Paul Dark^1^

*Research Group (data collection – alphabetical order*): Elena Apatri,^2^ Bethan Charles,^2^ Helen Christensen,^2^ Alice Harvey,^2^ Diane Lomas,^2^ Melanie Taylor,^2^ Vicky Thomas,^2^ Danielle Walker,^2^

^1^ Division of Immunology, Immunity to Infection and Respiratory Medicine, University of Manchester, Manchester, UK

^2^ Salford Royal NHS Foundation Trust, Salford, UK

**Nottingham University Hospitals NHS Trust**

*Principal Investigator*: Dominick Shaw^1^

*Research Group (data collection*): Lucy Howard,^2^ Amelia Joseph,^2^ Saheer Sultan^2^

^1^ Leicester NIHR Biomedical Research Centre and Department of Respiratory Sciences, University of Leicester, Leicester, UK

^2^ Nottingham University Hospitals NHS Trust, Nottingham, UK

**Patient and Public Representatives:**

Chikezie Knox-Macaulay^1^

Margaret Ogden^1^

Graham Prestwich^1^

Ryan Hamilton^2,3^

^1^ Centre for Trials Research, College of Biomedical and Life Sciences, Cardiff University, Cardiff, UK

^2^ Antibiotic Research UK, York, UK

^3^ School of Pharmacy, De Montfort University, Leicester, UK
