# Supplementary material for: Procalcitonin to guide antibiotic use during the first wave of COVID-19 in English and Welsh hospitals: integration and triangulation of findings from quantitative and qualitative sources
Source: BMJ Open. 2025 Aug 8;15(8):e093210. doi: 10.1136/bmjopen-2024-093210 (PMC12336465; doi:10.1136/bmjopen-2024-093210)
Supplement: online supplemental file 2 [file bmjopen-15-8-s002.pdf]

PEACH coding matrix

Findings from workstreams

Statement	Data Source 1: Survey (WP1.1)	Data Source 2: Organisational level data (WP1.2)	Data Source 3: Patient level data (WP2.1)	Data Source 4: Qualitative interviews (WP2.2)	Convergence coding
**1: During the first wave of the pandemic PCT testing reduced antibiotic prescribing**	**Agree:** Percieved value of PCT by the majority of responents 78/114 (68.4%)	**Agree:** ED/AMU: Introduction of PCT in emergency departments/acute medical admission units associated with an initial statistically significant decrease in total antibiotic use of -1.08 (95%CI: -1.81 to -0.36) defined daily doses (DDDs) of antibiotic per admission per week per trust.	**Agree:** PCT use was associated with reduced days of early antibiotics (within first 7 days of a positive COVID-19 test) and total days of antibiotic treatment.	**Agree:** Clinicians in hospitals where PCT was used previously or introduced during the first wave of the pandemic reported that the PCT test contributed to decision-making about antibiotic prescribing. They predicted that unnecessary antibiotic doses would have been reduced where the test was carried out. The stopping of antibiotics early was attributed to PCT results.	**Agreement**
**2: During the first wave of the pandemic PCT testing safely reduced antibiotic prescribing**	**No data**	**No data**	**Agree:** PCT testing was not associated with increased 30 or 60-day mortality and was not associated with an increase in hospital or intensive care unit length of stay.	**Partial agreement:** Most clinicians were positive about the use of PCT in guiding them to make antibiotic prescribing decisions. There was a divide between the majority who made the judgement that PCT had contributed safely to the reduction of antibiotic use within their hospital, and a minority who were more circumspect and would prefer to see evidence for the efficacy of PCT before it was used widely.	**Partial Agreement**
**3: PCT was not used as a central factor influencing antibiotic prescribing.**	**No data**	**Agree:** Although there was an initial significant drop in organisational prescribing, this declined over time. This effect was subsequently lost at a rate of 0.05 (95%CI: 0.02-0.08) DDDs per admission per week per Trust. Similar effects were found for first-line antibiotics prescribed for community-acquired pneumonia and for analysis restricted to COVID-19 admissions.	**No data**	**Agree:** During the first wave, there was a lot of confusion and rapidly changing advice around tests and treatments. Some clinicians reported that the tests contributed very little to the decision-making around antibiotic prescriptions, as these decisions were based on clinical judgement.	**Agreement**
**4: PCT testing reduced antibiotic prescribing in ED/AMU**	**No data**	**Agree:** Introduction of PCT in emergency departments/acute medical admission units (ED/AMU) was associated with an initial statistically significant decrease in total antibiotic use.	**No data**	**Agree:** Clinicians in EDs found that PCT use was more widespread than in other parts of the hospital. There was a heightened anxiety around the unknown infection at the beginning of the pandemic. This led to a need for more evidence for clinicians to be reassured in stopping antibiotics. Clinicians saw their role as providing evidence for de-escalation. PCT was seen as a useful tool for providing this evidence.	**Agreement**
**5: PCT testing reduced antibiotic prescribing in ICU**	**No data**	**Disagree:** In ICU settings, PCT was not associated with any significant change in antibiotic use.	**Disagree:** There was no statistically significant association between antibiotic prescribing in patients admitted early to ICU and baseline PCT testing.	**Agree:** Clinicians spoke about how PCT was used for reassurance purposes in ICU. As with ED, it was considered a useful tool to enable reduction of antibiotic use.	**Dissonance**
**6: There were many barriers to implementing PCT testing during the first wave of COVID-19**	**Partial agreement:** Fifty-five of 114 (48%) of respondents reported that their organisation had a guideline for PCT use.	**No data**	**No data**	**Agree:** During the first wave of the pandemic, clinicians reported a lot of confusion and rapidly changing advice and guidelines around tests and treatments, meaning that guidelines were not always followed. There was therefore chaotic implementation of PCT testing, with some clinicians being aware of guidelines and others not, and with guidelines being followed differently in different parts of the hospital.	**Partial Agreement**
**7: Local PCT guidelines/protocols were perceived to be valuable**	**No data**	**No data**	**No data**	**Agree:** Clinicians who reported using the available guidelines said that they were helpful, especially due to COVID being a new condition. This was particularly when the guidelines were very clear in the parameters, and when they were readily available to access.	**Silence**
