# Supplementary material for: Procalcitonin to guide antibiotic use during the first wave of COVID-19 in English and Welsh hospitals: integration and triangulation of findings from quantitative and qualitative sources
Source: BMJ Open. 2025 Aug 8;15(8):e093210. doi: 10.1136/bmjopen-2024-093210 (PMC12336465; doi:10.1136/bmjopen-2024-093210)
Supplement: online supplemental file 3 [file bmjopen-15-8-s003.pdf]

CONSENT SCRIPT FOR QUALITATIVE STUDY

Please listen carefully to the following statements and say ‘yes’ or ‘no’ after each statement to confirm whether you agree.

1.	I confirm that I have read and understood the Information Sheet for Qualitative Study (version 1.1 dated 24/02/2021) for the PEACH Qualitative Study. I have had the opportunity to consider the information, ask questions and have had these answered satisfactorily.	
2.	I understand that my participation is voluntary and that I am free to withdraw at any time, without giving any reason, without my legal rights being affected.	
3.	I am willing to talk with a member of the research team about my experiences and views on the diagnosis, management and treatment of patients with COVID-19 and antibiotic usage.	
4.	I give permission for my interview with the researcher to be audio-recorded.	
5.	I understand that information collected about me during the interview will be treated with the strictest confidentiality and may be listened to by the research team and an external transcription company. The recording will not be labelled with my name.	
6.	I understand that what I say in the interview may be quoted word for word in publications, presentations and reports, but the quote will be anonymous.	
7.	I understand that information collected about me will be held at the Centre for Trials Research, Cardiff University according to the UK GDPR, the retained EU law version of the General Data Protection Regulation ((EU) 2016/679). I understand that this information will be kept strictly confidential and that no personal information will be used in the study report or publications.	
8.	I agree to regulatory authorities and University of Leeds (sponsor) accessing the data obtained in this study where it is relevant to my taking part in research, on the understanding that all data will remain confidential.	
9.	I agree to take part in the above Qualitative Study.	

Cardiff University Centre for Trials Research / Prifysgol Caerdydd Canolfan Ymchwil Treialon

University of Leeds

Funded by NIHR | National Institute for Health Research

PEACH

Procalcitonin: Evaluation of Antibiotic use in COVID-19 Hospitalised Patients

Interview Topics Overview (Health Care Professionals)

The topic guide will include overarching topics we would like to cover, but will be flexible and allow the interview to be guided by interviewee in terms of order and wording, and allow the interviewee to initiate and develop topics that have not been pre-empted in advance.

INTRODUCTION:

Thank you for taking the time to speak to me today. My name is Josie. I'm not a clinician or a health professional, I'm a qualitative researcher at the Centre for Trials Research at Cardiff University. The sort of research I do involves speaking to people – often patients and health professionals – to find out about their feelings and experiences, ask them what is important to them, and ask them to describe it in their own words. I'm interested to hear what your views on the challenges and issues you faced and still face treating patients with suspected COVID and particularly about antibiotic use.

I would like to hear about your experiences and your opinions in your own words and there are no right or wrong answers. Anything you say will be really helpful. If you need to stop the interview then please feel free to say so at any time. Do you have any questions before we get started?

Consent script

Could you talk to me about your role (or your role from last year April 2020 to July 2020 if it's changed), for instance, what sort of hospital, ward, patients do you work with?

How long have you been in your role and how senior are you?

A. Antibiotic decision making

1. Extent of first wave of COVID-19 in your hospital – our dates are April 2020 to July 2020, but yours might be different – what was it like?

2. How did you decide whether patients hospitalised with COVID-19 needed to start antibiotics?

3. How do you decide which antibiotics to prescribe?

4. How do you decide when to stop or review antibiotics?

5. What clinical factors influenced your decision to start or stop or change antibiotics? e.g type/severity/duration of symptoms, follow on Q: do laboratory/test results have a role?

Or How important do you think laboratory or test results were to antibiotic prescribing decisions in COVID-19?

What impact would time to receive test results have on your decision-making? e.g. bedside test or sent to lab?

6. What non-clinical factors influenced your decision to start or stop or change antibiotics? e.g family, ethnicity, social support, home environment, late presentation, space on the ward(s), clinician's personal attitude to risk, previous experience, second opinion

B. Clinical guidelines (NB centres might have 'pathways', or 'algorithms'; these may be built into guidelines) [Explain 'guidelines' will be used to encompass all these resources].

1. In your hospital, did you routinely use a guideline to assist the decision to start or stop antibiotics in patients with COVID-19?

2. Guidelines: How useful were the guidelines? Did you overrule them? How often? What prompted you to do this? Did you consult more than one guideline?
   - How did you combine the guidelines with your own clinical judgement? How did you use guidelines to make decisions?
   - How could you improve the guidelines?
   - How early were the guidelines introduced? How was it shared and disseminated throughout clinical teams? Did your use of the guideline change over time?

Did your guideline/practice involve procalcitonin testing?

3. PCT

If yes, PCT test used during first wave

- How did you use the PCT test? E.g what part of the hospital? ICU, emergency department? Mainstream ward?
- Did you find the PCT test useful? What were the advantages of using the test?
- Has the extent to which you find it useful changed over the course of pandemic, or how confident are you in using compared to before?
- What were the disadvantages of using the test? What were the barriers to using the test?
- What is your personal experience of using PCT test before COVID-19? If PCT test only introduced during COVID-19: Can you imagine using the PCT test in everyday practice, after pandemic?
- Was use of PCT included in guidelines?
- Algorithm guidelines: How useful were the guidelines produced by the algorithm? Did you agree with the way in which the algorithm interpreted the PCT test results? Did you interpret the test results in a different way to the algorithm? Did you overrule the test results? How often? When?
- How did you combine the test result with your own clinical judgement? How did you use the test result to make decisions?
- What were patients' perceptions of using the PCT test? Did you use the test results and/or guidelines in discussions with patients?
- Do you think the use of PCT testing safely reduced antibiotic use among patients hospitalised with COVID-19? Where there other benefits in terms of antibiotic use?

If no PCT test and/or algorithm not used during first wave

- Do you think the introduction of (or more frequent/accessible?) PCT testing would be useful to support your clinical decision to stop and start antibiotics in patients with suspected COVID? Where do you think PCT could be most useful?
- Do you think the introduction of algorithm would be useful to support your clinical decision to stop and start antibiotics in patients with suspected COVID? Would you use one if it were introduced? What features do you think would be useful?
- Do you think the use of PCT testing could safely reduce antibiotic use among patients hospitalised with COVID-19?

NICE guidelines

What impact did NICE COVID-19 rapid guideline have on your Trust's decision about PCT use?

Attitude to PCT generally

Has your view of PCT changed during COVID-19?

Lessons learned?

- Thinking back in time to the busiest time during the first wave of COVID in spring 2020, do you think there are things that could have improved your practice and management of antibiotic use in patients?
- Are there lessons you learned that you took with you as COVID cases increased during winter 2020 and beyond? Have you noticed changes in your colleagues' thinking and behaviours?

Scenario: I'm going to share with you a case study with complexities where decision making is not straight forward. I'll then ask you to describe what your decision making would be around starting and stopping antibiotics.

78 years old female, white, BMI 18, admitted this morning

Unconfirmed COVID-19, awaiting test results

Symptoms started 3 days ago: fever (39.2C), loss of taste and diarrhoea

No significant shortness of breath, but respiratory rate increased to 22/min

SpO2 91% without oxygen. CXR shows bilateral pulmonary infiltrates – typical of COVID-19. Bloods: neutrophilia and raised CRP, elevated urea and creatinine (previously normal), Hb 92

PMHx: ischaemic heart disease, hypertension, renal impairment with reduced eGFR, known microcytic anaemia on Fe supplement

Explore with clinician how events and decisions occurred over time.
What clinical factors influenced your decision on starting and stopping antibiotics?
What other (non-clinical) factors influenced your decision on starting and stopping antibiotics?
How might this differ with a less complex patient case?

Then add:

Patient does not handle anti-biotics well.
History of C Difficile

Family involvement

Closing

- Is there anything you'd like to add that you think is relevant or important that you haven't had a chance to bring up yet?
