# Supplementary material for: Procalcitonin to guide antibiotic use during the first wave of COVID-19 in English and Welsh hospitals: integration and triangulation of findings from quantitative and qualitative sources
Source: BMJ Open. 2025 Aug 8;15(8):e093210. doi: 10.1136/bmjopen-2024-093210 (PMC12336465; doi:10.1136/bmjopen-2024-093210)
Supplement: Supplementary file 4 [file bmjopen-2024-093210.reviewer_comments.pdf]

PEER REVIEW HISTORY

BMJ Open publishes all reviews undertaken for accepted manuscripts. Reviewers are asked to complete a checklist review form and are provided with free text boxes to elaborate on their assessment. These free text comments are reproduced below.

ARTICLE DETAILS

Title (Provisional)

Procalcitonin to guide antibiotic use during the first wave of COVID-19 in English and Welsh hospitals: Integration and triangulation of findings from quantitative and qualitative sources

Authors

Henley, Josie; Brookes-Howell, Lucy; Howard, Philip; Powell, Neil; Albur, Mahableshwar; Bond, Stuart E; Euden, joanne; Dark, Paul; Grozeva, Detelina; Hellyer, Thomas P; Hopkins, Susan; Llewelyn, Martin; Maboshe, Wakunyambo; McCullagh, Iain J; Ogden , Margaret; Pallmann, Philip; Parsons, Helena K; Partridge, David G; Shaw, Dominick; Shinkins, Bethany; Szakmany, Tamas; Todd, Stacy; West, Robert M; Thomas-Jones, Emma; Carrol, Enitan; Sandoe, Jonathan; Cardiff, Peach

---

VERSION 1 - REVIEW

**Reviewer**	**1**
**Name**	**Zanolin, Maria Elisabetta**
**Affiliation**	**University of Verona, Unit of Epidemiology and Medical Statistics, Dept. Public Health and Community Medicine**
**Date**	**10-Dec-2024**
**COI**	**None**

The study topic is interesting, but there are some major points that should be addressed.

Strengths and limitations

Page 6, lines 8-10: On which findings is the affirmation “Investigator triangulation… reduces bias” based on?

Methods

Page 8, lines 21-31: How were the 6 out of 11 sites chosen? Randomly? Did the potential participants voluntarily decide to participate? Did you consider a potential selection bias?

Results

Page 11, Lines 18-25: “Data source 1” It is not clear where the results reported here are taken from, as ref 10 of Euden et al presents only a study protocol and no other references are cited at this point.

Page 11, Lines 29-36: ”Data source 2” The reference paper by Llewelyn et al should be cited here. In this paper it is also said that “This effect (of a statistically significant decrease in total antibiotic use) was then lost at a rate of 0.05 (95% CI: 0.02–0.08) DDDs per admission per week.” So can be this really considered as “agree”? With respect to which period the “initial … decrease in total antibiotic use” is referred to?

Page 11, Lines 40-50: “Data source 3” Where the results reported here can be find? Are they in press? If so, there is no possibility to check them.

Page 13, lines 27-33: Data source 3: Where the data on mortality can be find? Is there any reference?

Page 13, lines 37-47: Data source 4: Is the “partial agreement” based only on the clinicians’ opinions?

In Discussion and Conclusions, it should be kept into account that for some statements data from only two/three sources were available. In addition, the study should highlight studies that have emphasized the bacteriophage aspect of SARS-CoV-2. Such studies lend support to the use of the PCT biomarker, and its increase during COVID-19 infection is a critically important element that cannot be underestimated.

Reference 12 is not complete.

**Reviewer**	**2**
**Name**	**Karakoc , Hanife Nur**
**Affiliation**	**University of Maryland Baltimore School of Medicine**
**Date**	**27-Jan-2025**
**COI**	**None**

This is a valuable study in demonstrating the potential of procalcitonin as a guide for antibiotic use.

Dear editor,

I have reviewed the manuscript entitled " Procalcitonin to guide antibiotic use during the first wave of COVID-19 in English and Welsh hospitals: Integration and triangulation of findings from quantitative and qualitative sources”. My suggestions and comments are below:

1. **Patients and methods section**

It would benefit from clearly defining the terms co-infection and secondary infection. Providing these definitions would enhance the clarity and interpretation of the study's findings.

2. **Patients and methods section**
   The inclusion criteria for the patients should be clearly specified.
3. **In the results section**
   It would be helpful to clarify in which patients procalcitonin levels were elevated. Were patients with conditions that could complicate the interpretation of procalcitonin levels, such as elevated creatinine levels or malignancies, excluded from the study?
4. **Discussion section**
   It is recommended to include a paragraph discussing how procalcitonin levels are influenced by the severity of COVID-19 and its impact on antibiotic use in these patients. This addition would provide valuable insight into the relationship between procalcitonin dynamics and disease severity, further enhancing the study's relevance.

VERSION 1 - AUTHOR RESPONSE

	**Comment**	**Response (notes) comments in red are where I still need help**
	**Reviewer: 1**<br>Dr. Maria Elisabetta Zanolin, University of Verona	
1	Strengths and limitations<br>Page 6, lines 8-10: On which findings is the affirmation "Investigator triangulation... reduces bias" based on?	We agree that this comment may be confusing and have therefore changed the text to: "enriches the analysis and improves validity"
2	Methods<br>Page 8, lines 21-31: How were the 6 out of 11 sites chosen? Randomly? Did the potential participants voluntarily decide to participate? Did you consider a potential selection bias?	The following text has been added: "Sites were invited to participate based on their routine clinical use of PCT testing before and during the pandemic to ensure sites were included that did not use PCT, that did use PCT and that introduced PCT during the first wave (14). Prospective sampling was conducted with an aim to recruit participants with varied experience and roles within each site."<br><br>Sites were selected to ensure sites with different perspectives were included to reduce the risk of bias.

		Highlighted text has been added for clarity:<br>"Potential participants volunteered to participate and returned a consent to contact form and were contacted by the PEACH qualitative researcher to arrange an interview."<br><br>Participants did voluntarily decide to participate. The 'consent to contact' form is necessary due to UK data protection law so that only those who volunteered to participate would be contacted for interview. As such, participants were self-selected so there is the potential for selection bias, but this is no different from participants volunteering for interviews in any other trial.
3	Results<br>Page 11, Lines 18-25: "Data source 1" It is not clear where the results reported here are taken from, as ref 10 of Euden et al presents only a study protocol and no other references are cited at this point.	Reference 10 is Powell N, Howard P, Llewelyn MJ, Szakmany T, Albur M, Bond SE, et al. Use of Procalcitonin during the First Wave of COVID-19 in the Acute NHS Hospitals: A Retrospective Observational Study. Antibiotics (Basel). 2021 May 1;10(5):516. This reports the results for data source 1.<br><br>Euden et al protocol is ref 13.<br><br>Please note: The numbering of some references has changed in the revision due to including new references for recently published papers. However, the references up to 13 have remained as they were in the original manuscript.
4	Page 11, Lines 29-36: "Data source 2" The reference paper by Llewelyn et al should be cited here. In this paper it is also said that "This effect (of a statistically significant decrease in total antibiotic use) was then lost at a rate of 0.05 (95% CI: 0.02–0.08) DDDs per admission per week." So can be this really considered as "agree"? With	Within the limitations of the methods in this study, it is unequivocal that PCT use reduced antibiotic use, so we stand by our categorisation of "agree". If the research question had been: "Did PCT result in a sustained reduction in antibiotic use?" We would have to change our categorisation, but the fact that the reduction was slowly eroded is a separate issue.

	respect to which period the “initial … decrease in total antibiotic use” is referred to?	References have been added to findings as suggested.
5	Page 11, Lines 40-50: “Data source 3” Where the results reported here can be find? Are they in press? If so, there is no possibility to check them.	Data source 3 is now published and citation included as reference 14.<br><br>Sandoe JAT, Grozeva D, Albur M, Bond SE, Brookes-Howell L, Dark P, et al. A retrospective propensity-score-matched cohort study of the impact of procalcitonin testing on antibiotic use in hospitalized patients during the first wave of COVID-19. J Antimicrob Chemother. 2024 Nov 4;79(11):2792–800.
6	Page 13, lines 27-33: Data source 3: Where the data on mortality can be find? Is there any reference?	Data source 3 is now published and citation included as above.
7	Page 13, lines 37-47: Data source 4: Is the “partial agreement” based only on the clinicians’ opinions?	Yes. Added text to the opening paragraph of finding 2. “This partial agreement is based on clinicians’ opinions.”
8	In Discussion and Conclusions, it should be kept into account that for some statements data from only two/three sources were available.	We agree, text added to the discussion as suggested “It should be noted that for some statements, not all data sources provided data.”
9	In addition, the study should highlight studies that have emphasized the bacteriophage aspect of SARS-CoV-2. Such studies lend support to the use of the PCT biomarker, and its increase during COVID-19 infection is a critically important element that cannot be underestimated.	We apologise but we don’t understand this comment and have therefore not made any changes related to this request. Please get back to us if needed and we can draft a response.
10	Reference 12 is not complete.	Apologies, the Webb et al reference has been updated. This is now reference 19 due to revisions.
	**Reviewer: 2**<br>Dr. Hanife Nur Karakoc , University of Maryland Baltimore School of Medicine	
1	Patients and methods section	We have added the following text to the introduction and referenced to Langford

	It would benefit from clearly defining the terms co-infection and secondary infection. Providing these definitions would enhance the clarity and interpretation of the study's findings.	et al. (2020) for clarity. "A distinction is drawn between bacterial co-infection (present at presentation) and secondary bacterial infection (which develops after presentation) in patients with COVID-19 (4)."
2	Patients and methods section<br>The inclusion criteria for the patients should be clearly specified.	The following text has been added to the 'Setting and participants' section of methods for data source 3:<br><br>"Inclusion criteria: patients ≥16 years, admitted to participating Trusts/Health Boards and with a confirmed positive COVID-19 test between 1 February 2020 and 30 June 2020."
3	In the results section<br>It would be helpful to clarify in which patients procalcitonin levels were elevated. Were patients with conditions that could complicate the interpretation of procalcitonin levels, such as elevated creatinine levels or malignancies, excluded from the study?	If we have understood correctly, the reviewer is asking about confounding factors, because certain patients may be more likely to have a PCT test and certain conditions may increase PCT other than infection. (NB These are covered in detail in ref 14.) We have added the following text to the methods section as we feel this is a methodological issue rather than a result:<br><br>"We utilised propensity score matching in data source 3 to ensure an even distribution of important confounders between the tested groups. Propensity-score matching was used to reduce the potential differences between the 'tested' (i.e. PCT test at baseline) and 'untested' patients (i.e. no PCT test at baseline) as described in the main paper for this data source (14)."
4	Discussion section<br>It is recommended to include a paragraph discussing how procalcitonin levels are influenced by the severity of COVID-19 and its impact on antibiotic use in these patients. This addition	The following text has been added to the discussion, strengths and limitations:<br>"When considering the source data, it should be noted that a study of viral pneumonia completed before COVID-19, found low mortality (1.6%) but that patients had a range of peak PCT values, indicating that viral infection can

	would provide valuable insight into the relationship between procalcitonin dynamics and disease severity, further enhancing the study's relevance.	increase PCT (29). PCT did not distinguish well between pure viral pneumonia and bacterial co-infection in this study, but performed better as a prognostic marker (29). An association between raised PCT (>0.5ng/mL) and poor prognosis in COVID-19 was noted early in the pandemic (30). This is relevant because PCT may have been carried out, either consciously or unconsciously, in more unwell patients, meaning that severity of illness is a potentially confounding factor in any analysis of PCT in COVID-19. This highlights why propensity score matching was necessary to reduce the risk of bias in the analysis of data source 3 and why there is a risk of bias in perspectives about the role of PCT."<br><br>References 29 and 30 have been added.
	**Additional Amendments**	
		Checked formatting of all references, up to date and including new references, checked reference numbers in this table correspond to the revised manuscript.

VERSION 2 - REVIEW

Reviewer 1

Name Zanolin, Maria Elisabetta

Affiliation University of Verona, Unit of Epidemiology and Medical Statistics, Dept. Public Health and Community Medicine

Date 24-Apr-2025

COI

I would like to address here a comment that was not understood by the authors: There are some studies showing that SARS-CoV-2 is also a bacteriophage (i.e. Brogna et al, J Med Virol, 2024, 96(3): e29507) and that, as a consequence, an early use of specific combinations of antibiotics at home reduces disease duration, maintains higher levels of blood oxygen saturation in the patients and protects from long COVID. Therefore, I think it is important that you specify in the limits of the study that, in your triangulation, you have considered

only hospitalized patients, that you have not considered which specific antibiotics have been used and that you have not taken into account the consequences on long COVID. So, for equality police and transparence in the scientific publication, it is important that you explain the limitations of your triangulation study.

This and the other comments are in the attached file.

	**Comment**	**Response (notes) comments in red are where I still need help**
	**Reviewer: 1**<br>Dr. Maria Elisabetta Zanolin, University of Verona	
1	Strengths and limitations<br>Page 6, lines 8-10: On which findings is the affirmation "Investigator triangulation… reduces bias" based on?<br>*ok, thank you*	We agree that this comment may be confusing and have therefore changed the text to: "enriches the analysis and improves validity"
2	Methods<br>Page 8, lines 21-31: How were the 6 out of 11 sites chosen? Randomly? Did the potential participants voluntarily decide to participate? Did you consider a potential selection bias?<br><br>*Ok, thank you for the explanation. Anyway, the possible volunteer bias should be included in the limits*	The following text has been added: "Sites were invited to participate based on their routine clinical use of PCT testing before and during the pandemic to ensure sites were included that did not use PCT, that did use PCT and that introduced PCT during the first wave (14). Prospective sampling was conducted with an aim to recruit participants with varied experience and roles within each site."<br><br>Sites were selected to ensure sites with different perspectives were included to reduce the risk of bias.<br><br>Highlighted text has been added for clarity:<br>"Potential participants volunteered to participate and returned a consent to contact form and were contacted by the PEACH qualitative researcher to arrange an interview."<br><br>Participants did voluntarily decide to participate. The 'consent to contact' form is necessary due to UK data protection law so that only those who volunteered to participate would be contacted for interview. As such, participants were self-

		selected so there is the potential for selection bias, but this is no different from participants volunteering for interviews in any other trial.
3	Results<br>Page 11, Lines 18-25: "Data source 1" It is not clear where the results reported here are taken from, as ref 10 of Euden et al presents only a study protocol and no other references are cited at this point.<br><br>*ok, thank you*	Reference 10 is Powell N, Howard P, Llewelyn MJ, Szakmany T, Albur M, Bond SE, et al. Use of Procalcitonin during the First Wave of COVID-19 in the Acute NHS Hospitals: A Retrospective Observational Study. Antibiotics (Basel). 2021 May 1;10(5):516. This reports the results for data source 1.<br><br>Euden et al protocol is ref 13.<br><br>Please note: The numbering of some references has changed in the revision due to including new references for recently published papers. However, the references up to 13 have remained as they were in the original manuscript.
4	Page 11, Lines 29-36: "Data source 2" The reference paper by Llewelyn et al should be cited here. In this paper it is also said that "This effect (of a statistically significant decrease in total antibiotic use) was then lost at a rate of 0.05 (95% CI: 0.02–0.08) DDDs per admission per week." So can be this really considered as "agree"? With respect to which period the "initial ... decrease in total antibiotic use" is referred to?<br><br>*Thank you. Anyway, the period to which the statistically significant decrease in total antibiotic use is referred should be specified in the text, as the statement "During the first wave of the pandemic PCT testing reduced antibiotic prescribing" is general and not linked to a particular lapse of time.*	Within the limitations of the methods in this study, it is unequivocal that PCT use reduced antibiotic use, so we stand by our categorisation of "agree". If the research question had been: "Did PCT result in a sustained reduction in antibiotic use?" We would have to change our categorisation, but the fact that the reduction was slowly eroded is a separate issue.<br><br>References have been added to findings as suggested.

5	Page 11, Lines 40-50: "Data source 3" Where the results reported here can be find? Are they in press? If so, there is no possibility to check them.<br><br>*ok, thank you*	Data source 3 is now published and citation included as reference 14.<br><br>Sandoe JAT, Grozeva D, Albur M, Bond SE, Brookes-Howell L, Dark P, et al. A retrospective propensity-score-matched cohort study of the impact of procalcitonin testing on antibiotic use in hospitalized patients during the first wave of COVID-19. J Antimicrob Chemother. 2024 Nov 4;79(11):2792–800.
6	Page 13, lines 27-33: Data source 3: Where the data on mortality can be find? Is there any reference?<br><br>*ok, thank you*	Data source 3 is now published and citation included as above.
7	Page 13, lines 37-47: Data source 4: Is the "partial agreement" based only on the clinicians' opinions?<br><br>*ok, thank you*	Yes. Added text to the opening paragraph of finding 2. "This partial agreement is based on clinicians' opinions."
8	In Discussion and Conclusions, it should be kept into account that for some statements data from only two/three sources were available.<br><br>*ok, thank you*	We agree, text added to the discussion as suggested "It should be noted that for some statements, not all data sources provided data."
9	In addition, the study should highlight studies that have emphasized the bacteriophage aspect of SARS-CoV-2. Such studies lend support to the use of the PCT biomarker, and its increase during COVID-19 infection is a critically important element that cannot be underestimated.<br><br>*I clarify my comment below:*<br>*There are some studies showing that SARS-CoV-2 is also a bacteriophage (i.e. Brogna et al, J Med Virol, 2024, 96(3): e29507) and that, as a consequence, an early*	We apologise but we don't understand this comment and have therefore not made any changes related to this request. Please get back to us if needed and we can draft a response.

	use of specific combinations of antibiotics at home reduces disease duration, maintains higher levels of blood oxygen saturation in the patients and protects from long COVID. Therefore, I think it is important that you specify in the limits of the study that, in your triangulation, you have considered only hospitalized patients, that you have not considered which specific antibiotics have been used and that you have not taken into account the consequences on long COVID. So, for equality police and transparence in the scientific publication, it is important that you explain the limitations of your triangulation study.	
10	Reference 12 is not complete.<br><br>*ok, thank you*	Apologies, the Webb et al reference has been updated. This is now reference 19 due to revisions.

VERSION 2 - AUTHOR RESPONSE

	Comment	Response
	**Reviewer: 1**<br>Dr. Maria Elisabetta Zanolin, University of Verona	
1	Methods<br>Page 8, lines 21-31: How were the 6 out of 11 sites chosen? Randomly? Did the potential participants voluntarily decide to participate? Did you consider a potential selection bias?<br><br>*Ok, thank you for the explanation. Anyway, the possible volunteer bias should be included in the limits*	Following the previous revision, we have included possible volunteer bias in the limitations.

2	Page 11, Lines 29-36: "Data source 2" The reference paper by Llewelyn et al should be cited here. In this paper it is also said that "This effect (of a statistically significant decrease in total antibiotic use) was then lost at a rate of 0.05 (95% CI: 0.02–0.08) DDDs per admission per week." So can be this really considered as "agree"? With respect to which period the "initial ... decrease in total antibiotic use" is referred to?<br><br>*Thank you. Anyway, the period to which the statistically significant decrease in total antibiotic use is referred should be specified in the text, as the statement "During the first wave of the pandemic PCT testing reduced antibiotic prescribing" is general and not linked to a particular lapse of time.*	Following the previous revision, we have added dates to the two statements that make reference to the first wave, 01/02/2020-30/06/2020 from the inclusion criteria for Data source 3.
3	In addition, the study should highlight studies that have emphasized the bacteriophage aspect of SARS-CoV-2. Such studies lend support to the use of the PCT biomarker, and its increase during COVID-19 infection is a critically important element that cannot be underestimated.<br><br>*I clarify my comment below: There are some studies showing that SARS-CoV-2 is also a bacteriophage (i.e. Brogna et al, J Med Virol, 2024, 96(3): e29507) and that, as a consequence, an early use of specific combinations of antibiotics at home reduces disease duration, maintains higher levels of blood oxygen saturation in the patients and protects from long COVID. Therefore, I think it is important that you specify in the*	We did not take into account any antibiotics prescribed to patients prior to admission. We have incorporated this point into our limitations section. Thank you for highlighting this.

	limits of the study that, in your triangulation, you have considered only hospitalized patients, that you have not considered which specific antibiotics have been used and that you have not taken into account the consequences on long COVID. So, for equality police and transparence in the scientific publication, it is important that you explain the limitations of your triangulation study.	
